# High Incidence of Isolated Tumor Cells in Sentinel Node Biopsies of Thin Melanomas: A Potential Factor in the Paradoxical Prognosis of Stage IIIA Cutaneous Melanoma?

**DOI:** 10.3390/diagnostics15010069

**Published:** 2024-12-30

**Authors:** Andrea Ronchi, Giuseppe D’Abbronzo, Emma Carraturo, Giuseppe Argenziano, Gabriella Brancaccio, Camila Scharf, Elvira Moscarella, Teresa Troiani, Francesco Iovino, Salvatore Tolone, Mario Faenza, Gerardo Cazzato, Renato Franco

**Affiliations:** 1 Pathology Unit, Department of Mental and Physical Health and Preventive Medicine, University of Campania Luigi Vanvitelli, 81100 Naples, Italy; andrea.ronchi@unicampania.it (A.R.); dabbronzogiuseppe@gmail.com (G.D.); em.carraturo@gmail.com (E.C.); 2 Dermatology Unit, Department of Mental and Physical Health and Preventive Medicine, University of Campania Luigi Vanvitelli, 81100 Naples, Italy; giuseppe.argenziano@unicampania.it (G.A.); gabriella.brancaccio@unicampania.it (G.B.); camila.araujoscharfpinto@unicampania.it (C.S.); elvira.moscarella@unicampania.it (E.M.); 3 Medical Oncology Unit, Department of Precision Medicine, University of Campania Luigi Vanvitelli, 81100 Naples, Italy; teresa.troiani@unicampania.it; 4 Department of Translational Medicine Science, School of Medicine, University of Campania Luigi Vanvitelli, 81100 Naples, Italy; francesco.iovino@unicampania.it; 5 Department of Advanced Medical and Surgical Sciences, University of Campania Luigi Vanvitelli, 81100 Naples, Italy; salvatore.tolone@unicampania.it; 6 Plastic Surgery Unit, Multidisciplinary Department of Medical-Surgical and Dental Specialties, University of Campania Luigi Vanvitelli, 81100 Naples, Italy; mario.faenza@unicampania.it; 7 Section of Molecular Pathology, Department of Precision and Regenerative Medicine and Ionian Area (DiMePRe-J), University of Bari “Aldo Moro”, 70121 Bari, Italy; gerycazzato@hotmail.it

**Keywords:** sentinel node biopsy, cutaneous melanoma, isolated tumor cells, staging, prognosis

## Abstract

**Background/Objectives**: This study aims to evaluate whether the presence of isolated tumor cells (ITCs) correlates with specific stages of cutaneous melanoma, potentially shedding light on their prognostic significance and the paradoxical survival outcomes in stage IIIA. **Methods**: This study analyzed cases of sentinel lymph node biopsies for cutaneous melanoma between 2021 and 2023. It included patients with CM diagnoses, available histological slides, and clinical information about the neoplasia stage. The correlation between the primary tumor stage and the presence of isolated tumor cells was statistically analyzed. **Results**: This study analyzed 462 sentinel lymph node biopsies, revealing 77.1% negative cases and 22.9% positive cases. Isolated tumor cells were observed in 24 cases (5.2%), most commonly in the early stages (e.g., pT1b and pT2a). Statistical analysis confirmed a significant correlation between ITC presence and early-stage neoplasms (*p* = 0.014). **Conclusions**: Although ITCs prompt upstaging, their prognostic impact appears limited, especially in thin melanomas, where survival aligns more closely with stage IB than stage IIIA. This aligns with findings from breast cancer studies where ITCs are not equated to metastases in staging due to their minimal impact on prognosis. Current melanoma staging practices could benefit from differentiating ITCs from larger metastatic deposits to better reflect the actual metastatic burden and guide treatment decisions.

## 1. Introduction

Cutaneous melanoma (CM) is currently the fifth most prevalent malignancy and one of the cancers with the greatest incidence increase over the past 50 years [1]. According to the latest SEER data, there were an estimated 100,640 new cases of CM in 2024 in the United States, accounting for 5.0% of all new cancer cases [2]. CM staging is the primary prognostic factor for this cancer, encompassing Breslow thickness, ulceration, and the presence of lymph node metastases and distant metastases. The management of the patient, therefore, primarily depends on the stage of the cancer. According to the AJJC 8th edition, 11 stage classes are defined, including 0, IA, IB, IIA, IIB, IIC, IIIA, IIIB, IIIC, IIID, and IV. Mortality strictly depends on stage, and the 5-year relative survival rates for localized (stages 0, I, and II), regional (stage III), and metastatic (stage IV) CMs are >99%, 74%, and 35%, respectively [3]. Although staging is the most important tool for predicting patient prognosis and consequently making appropriate clinical decisions, there seems to be a discrepancy in the current staging system when assessing prognosis: The survival rate for patients in stage IIIA is paradoxically higher than that for patients in a lower stage, specifically stage IIC [4,5]. Thus, patients with a high Breslow thickness and no lymph node metastases (stage IIC) have a worse prognosis compared to those with a lower Breslow thickness but with lymph node metastases (stage IIIA). Indeed, the 5-year disease-specific survival (DSS) rates for stages IIIA and IIC are 93% and 82%, respectively [6].

Sentinel node biopsy (SNB) is indicated for all cases ranging from pT1b to pT4b and currently serves a primarily prognostic and staging role [7]. The histological examination of the sentinel lymph node is widely based on the protocol by Cook et al., based on the examination of multiple sections at various depths, stained with hematoxylin and eosin and complemented by immunohistochemistry [8]. This extensive examination of the lymph node parenchyma is highly sensitive and can even detect isolated tumor cells (ITCs) [8]. Notably, when evaluating a sentinel lymph node biopsy (SNB), the lymph node should be considered positive for metastasis even if only a few ITCs are present [9]. This assessment applies specifically to CM, whereas for other neoplasms, such as breast carcinoma, ITCs are not considered prognostically significant; therefore, their presence is not an indication that the lymph node is metastatic. Recent evidence suggests that ITCs also do not have a significant prognostic impact in thin melanomas [10]. So, it can be hypothesized that the paradoxically favorable outcome of stage IIIA may be due to the presence of cases of thin melanoma where only isolated cells are found in the sentinel lymph node.

The aim of this study is to examine a series of sentinel lymph nodes analyzed between 2021 and 2023 in order to investigate the frequency of ITCs in SNBs and whether they correlate with a specific stage of the neoplasm.

## 2. Materials and Methods

All cases of SNBs for CM managed at the University “Luigi Vanvitelli” Hospital (Naples, Italy) from 1 January 2021 to 31 December 2023 were obtained from the archives of the Pathology Unit. The inclusion criteria were as follows: (1) SNB performed for the diagnosis of CM; (2) availability of corresponding histological slides; and (3) availability of clinical information regarding the stage of the neoplasm. Written informed consent, including permission to utilize the diagnostic data for scientific purposes, was obtained from each patient. This study was conducted according to the guidelines of the Declaration of Helsinki and approved by the ethics committee of “Vanvitelli” University (protocol code 282; approval date 6 October 2020).

All cases of sentinel lymph nodes included in this study were processed according to the protocol defined by Cook et al. [8]. For the immunohistochemical staining, we substituted SOX10 for S100, as previously proposed by several authors, because of its higher specificity and equal sensitivity [11,12,13,14]. Immunohistochemistry was performed on 4 µm thick formalin-fixed and paraffin-embedded (FFPE) slices using a fully automatized assay on the Ventana^®^ Benchmark XT platform (Ventana-Roche Diagnostics, Meylan, France). The procedure was performed according to the manufacturer’s instructions.

All histological slides were retrieved from the archives, including both hematoxylin and eosin-stained slides and immunostained slides. Expert pathologists reviewed all the slides, assessing the presence or absence of metastases and the quantity of tumor present in the lymph node (single cells vs. metastatic aggregates).

Data on SNBs performed at our institution during the period 2019–2020, prior to the implementation of the protocol established by Cook et al., were retrieved from institutional archives. This retrospective analysis aimed to compare the frequency of ITCs between these cases and the samples under investigation.

The statistical correlation between the stage of the primary neoplasm and the presence of ITCs in the SNB was assessed using Spearman’s correlation, employing “IBM SPSS Statistics version 27” software. Values of *p* < 0.05 were considered statistically significant.

## 3. Results

### 3.1. Clinicopathological Features

The series included 462 patients affected by CM and submitted for SNBs. The clinicopathological features of the series are detailed in Table 1.

Overall, 356 (77.1%) cases were negative and 106 (22.9%) cases were positive, indicating the presence of neoplasia.

**Figure 1:**
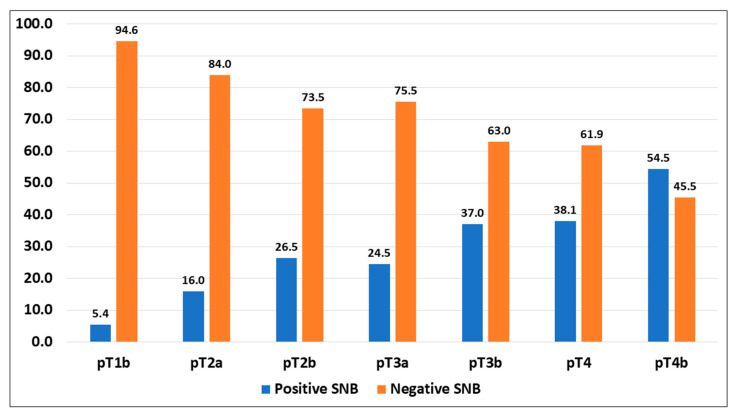
SNB assessment according to the stage.

Regarding the stage of the neoplasm, 112 cases were classified as pT1b; of these, in 6 cases, the SNBs (5.4%) were positive for neoplasia, while in the remaining 106 (94.6%) cases, the SNBs were negative. A total of 131 cases were classified as stage pT2a; of these, in 21 (16.0%) cases, SNBs were positive, and in the remaining 110 (84.0%) cases, the SNBs were negative. A total of 34 cases were classified as stage pT2b. In 9 (26.5%) cases, the SNBs were positive, and in 25 (73.5%) cases, the SNBs were negative. The pT3a stage included 52 cases, with 12 (23.1%) cases showing positive SNBs and 40 (76.9%) cases showing negative SNBs. A total of 54 cases were classified as pT3b; of these, in 20 (37.0%) cases, the SNBs were positive, and in 34 (63.0%) cases, the SNBs were negative. A total of 24 cases were classified as pT4a; in 8 (33.3%) cases, the SNBs were positive, while in 16 (66.7%) cases, the SNBs were negative. Lastly, 55 cases were classified as pT4b; of these, in 30 (54.5%) cases, the SNBs were positive, and in 25 (45.5%) cases, the SNBs were negative. The results are presented in Table 2 and Figure 1.

**Figure 2:**
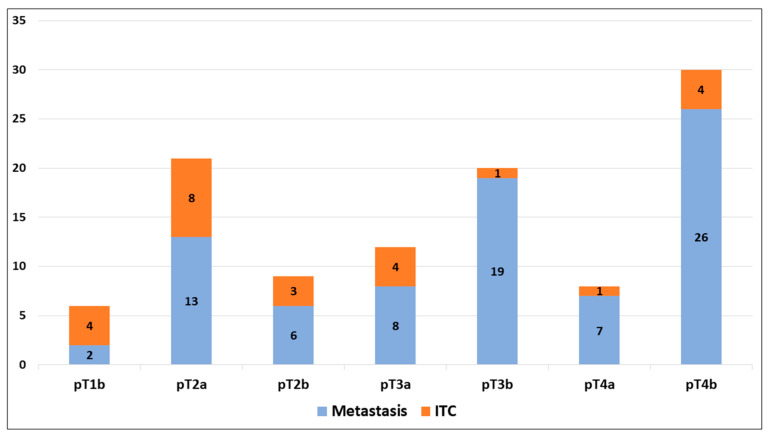
Assessment of metastases and isolated neoplastic cells (ITCs) in positive sentinel node biopsies according to the stage.

ITCs were observed in 24 out of 462 (5.2%) cases, including 4 cases in stage pT1b (66.7% of positive SNBs in stage pT1b; 3.8% of all SNBs), 8 cases in stage pT2a (38.1% of positive SNBs in stage pT2a; 7.5% of all SNBs), 3 cases in stage pT2b (33.3% of positive SNBs in stage pT2b; 2.8% of all SNBs), 4 cases in stage pT3a (33.3% of positive SNBs in stage pT3a; 3.8% of all SNBs), 1 case in stage pT3b (5.0% of positive SNBs in stage pT3b; 0.9% of all SNBs), 1 case in stage pT4a (12.5% of positive SNBs in stage pT1b; 0.9% of all SNBs), and 4 cases in stage pT4b (13.3% of positive SNBs in stage pT1b; 3.8% of all SNBs), as shown in Figure 2.

**Figure 3:**
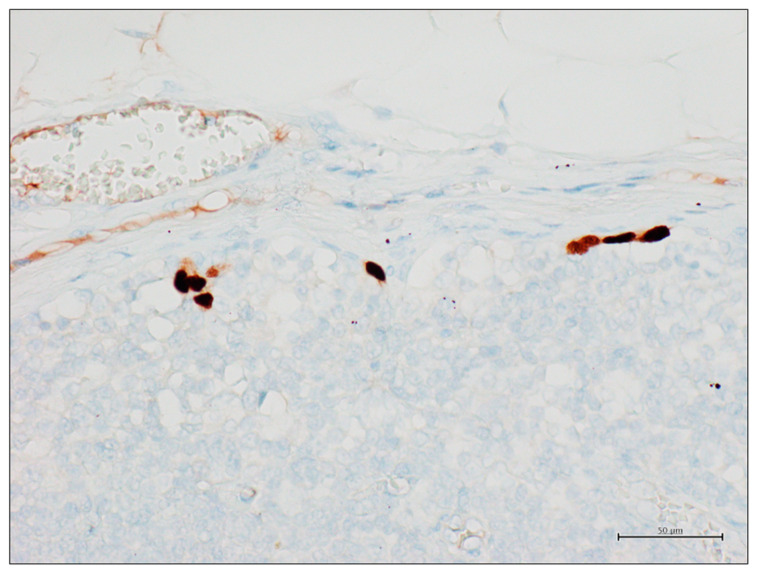
SOX10-positive isolated tumor cells in a sentinel node biopsy (SOX10 immunohistochemical stain, original magnification 200×).

An example of ITCs in an SNB sample is shown in Figure 3.

A total of 90 SNBs were performed at our institution in 2019 and 2020. Retrospective analysis of these cases revealed the presence of ITCs in only three cases, accounting for 3.3% of the total.

### 3.2. Statistical Analysis

Spearman’s correlation demonstrated a statistically significant correlation between the presence of ITCs in the sentinel lymph node and early stages of primary neoplasm (pT1b or pT2a), with an rs (Spearman’s Rank Correlation Coefficient) of 0.265 (*p*-value: 0.014).

## 4. Discussion

Sentinel node biopsy (SNB) is a cornerstone in the staging of cutaneous melanoma (CM). This procedure is carried out on the basis of the Breslow thickness of primary tumors, specifically for cases classified from pT1b to pT4b. Examination of SNB samples is worldwide based on the protocol proposed by Cook et al., including multiple histological sections stained by hematoxylin and eosin, complemented by immunohistochemistry, to ensure precise staging and assessment. SNB plays a critical role in determining the neoplasm stage, and its results significantly impact CM outcomes. Thin cutaneous melanomas (classified as pT1a, pT1b, and pT2a) with a positive SNB are assigned to stage IIIA, while thicker melanomas (pT3 and pT4) with a negative SNB result are classified as stages IIB and IIC (see Table 1). Importantly, in the evaluation of an SNB, the lymph node should be classified as positive for metastasis even if only a small number of ITCs are detected [9]. However, stage IIC patients typically have a worse prognosis than stage IIIA patients, with 5-year disease-specific survival (DSS) rates of 82% and 93%, respectively, suggesting that thickness rather than the SNB result impacts the prognosis more [6]. The paradoxical survival outcomes between stages IIC and IIIA may be explained by the interpretation that is currently given to ITCs detected in SNBs.

This study evaluated the presence of ITCs in SNBs from patients with cutaneous melanoma (CM) at various stages. ITCs were detected in 24 out of 462 (5.2%) cases, including 4 cases in stage pT1b, 8 cases in stage pT2a, 3 cases in stage pT2b, 4 cases in stage pT3a, 1 case in stage pT3b, 1 case in stage pT4a, and 4 cases in stage pT4b. A notable finding was the higher prevalence of ITCs in early-stage melanomas (pT1b and pT2a), as indicated by the statistically significant Spearman correlation (*p* = 0.014). The histological evaluation of sentinel lymph nodes typically adheres to the protocol outlined by Cook et al., which entails examining multiple tissue sections at varying depths. These sections are stained using hematoxylin and eosin and further analyzed through immunohistochemical methods. This meticulous approach to lymph node analysis proves highly effective in detecting metastases, and it also has a high sensitivity in detecting ITCs. In our institution, Cook’s protocol has been adopted since 2021, while previously SNBs were examined by hematoxylin and eosin staining and a single immunohistochemical staining for each block. Interestingly, 90 SNBs were examined in our institution in 2019 and 2020, and ITCs were detected only in 3 cases (3.3% of cases), while ITCs were detected in 5.2% (24 out of 462) of cases in the 2021–2023 period, when Cook’s protocol was applied, with an increase in positivity of 63.5%. Notably, two out of the three cases of ITCs detected in 2019–2020 were thin melanomas staged pT2a, confirming the data showing that ITCs are more frequently observed in cases of thin melanoma. These data support the hypothesis that ITCs have become particularly frequent in recent years, applying a very sensitive protocol. However, the real prognostic impact of ITCs in thin melanomas is not yet well defined. Although ITC-positive thin melanomas are classified as stage IIIA, the presence of ITCs in the sentinel lymph node may not have a significant prognostic impact, potentially undermining the rationale for assigning a higher stage. The case series analyzed in this study is relatively recent, as it is associated with the implementation of a relatively new protocol. Consequently, the prognostic evaluation is significantly limited by the overly short follow-up period, which limits the ability to perform a comprehensive multivariate statistical analysis. However, it is noteworthy that in our series, disease progression occurred in only one case of melanoma with ITCs. This case involved a patient with pT4b melanoma who declined adjuvant therapy and experienced lymph node progression and brain metastases within 8 months of diagnosis. On the other hand, disease progression was observed in six cases of thick melanomas (one case of pT3a, one case of pT4a, and four cases of pT4b) which presented a metastasis in the SNB. The progression occurred between 2 and 23 months after the diagnoses and involved lymph node metastases in all cases. In one instance, a cutaneous metastasis was also identified in addition to the lymph node metastasis. Regarding thin melanomas with ITCs (stage IIIA melanomas), no cases showed disease progression in a follow-up ranging from 1 to 3 years. Therefore, in our series, thin melanomas with ITCs, staged as IIIA, exhibited the same biological behavior as thin melanomas without ITCs. These data align with studies suggesting that ITCs in thin melanomas correlate with a prognosis similar to that of stage IB [10]. Thus, while the presence of ITCs traditionally leads to categorizing the lymph node as metastatic, it may not necessarily indicate a worse prognosis in the early stages. This supports the hypothesis that ITCs might represent a minimal metastatic burden with limited prognostic impact, especially in the context of thin CMs, where clinical outcomes remain favorable despite SNB positivity, implying that their detection should be considered cautiously in the staging process.

An important issue in CM staging involves understanding the biological and clinical implications of ITCs in SNBs compared to more extensive metastatic deposits. In other cancers, such as breast carcinoma, ITCs are not considered equivalent to metastatic disease and do not typically affect the staging classification. The presence of ITCs in breast cancer lymph nodes does not lead to upstaging, since studies have demonstrated that ITCs alone generally do not confer an increased risk of recurrence or poor outcomes. In contrast, lymph nodes with ITCs are currently classified as metastatic in melanoma staging, leading to upstaging and potentially overtreatment for patients whose prognosis might otherwise be favorable, as seen with patients in stage IIIA. In breast cancer, isolated tumor cells (ITCs) detected in lymph nodes have been a subject of extensive study, particularly regarding their impact on prognosis and implications for staging and treatment. ITCs are defined as single cells or small clusters not exceeding 0.2 mm and are commonly identified through immunohistochemical staining. Unlike more extensive lymph node metastases, ITCs are considered to represent a minimal tumor burden, and research has demonstrated that their presence does not significantly affect overall survival or recurrence rates in breast cancer. Several large studies have investigated the prognostic impact of ITCs in breast cancer lymph nodes, suggesting that these cells do not confer a worse prognosis when present in isolation. For example, the MIRROR study, including over 2700 patients with early-stage breast cancer, demonstrated that ITCs alone did not significantly impact disease-free survival when compared to patients with node-negative disease [15]. Thus, the presence of ITCs alone is not an indication of the same aggressive treatment strategies used in patients with larger metastatic deposits, since the risk of recurrence is minimal. Similarly, findings from the ACOSOG Z0010 trial, a large multicenter study of sentinel lymph node biopsies in breast cancer, demonstrated that patients with ITCs had no significant difference in survival outcomes compared to patients without nodal involvement [16]. This reinforces the notion that ITCs do not carry the same prognostic weight as larger nodal metastases, and that their presence does not address upstaging or intensified systemic therapy. The NSABP B-32 trial, another major study that included over 5000 breast cancer patients, also explored the clinical significance of ITCs and micrometastases in sentinel lymph nodes. The researchers found that micrometastases (0.2–2.0 mm) were associated with a slightly higher risk of recurrence than ITCs, but the overall impact on survival was modest [17]. This trial further supported the position that ITCs alone are not strong prognostic markers in breast cancer. As a result of these findings, current guidelines from the American Joint Committee on Cancer (AJCC) and the Union for International Cancer Control (UICC) recommend that ITCs in breast cancer lymph nodes should not lead to upstaging. The AJCC classifies ITCs separately from micrometastases and macrometastases, acknowledging their limited clinical impact. Accordingly, the detection of ITCs in breast cancer is typically recorded but does not alter staging or prompt the same treatment escalation that would be warranted for larger nodal metastases. The distinction between ITCs and larger metastatic deposits may be particularly relevant in melanoma, where staging relies on detecting nodal involvement as a critical prognostic indicator. The absence of a distinction between ITCs and larger metastatic foci in melanoma staging could therefore contribute to some of the paradoxical outcomes observed between stages IIC and IIIA. The clinical significance of this distinction also raises questions about how melanoma staging could evolve to better reflect the actual metastatic burden. If ITCs in SNBs do not contribute to poorer outcomes in patients with thin melanomas, differentiating them from more substantial metastases could refine staging, allowing for more accurate prognosis and treatment planning.

Several studies have examined the prognostic implications of isolated tumor cells (ITCs) in the sentinel lymph nodes (SLNs) of melanoma patients, emphasizing the correlation between lymph node tumor burden and survival outcomes. Research conducted by the Italian Melanoma Intergroup (IMI) indicated that a higher tumor burden in sentinel nodes correlates with poorer survival. Specifically, the study found that an increased metastatic deposit diameter in the sentinel nodes serves as a significant predictor of adverse prognosis. By integrating tumor burden with other variables such as ulceration and Breslow thickness, the multivariate models effectively stratified risk, thereby underscoring the prognostic relevance of tumor burden in SLN-positive melanoma patients [18]. Another investigation explored the prognostic utility of disseminated melanoma cell density within sentinel lymph nodes. Using immunocytology, the researchers quantified disseminated cancer cell (DCC) density and found a strong association between increased DCC density and elevated melanoma-specific mortality risk. This quantitative approach, leveraging metastatic cell counts, significantly enhanced predictive accuracy for patient outcomes beyond traditional histopathological measures [19]. Additionally, European research efforts, notably the EORTC-DeCOG, have developed nomograms to predict recurrence and survival outcomes based on SLN tumor burden, patient age, and other clinical parameters. Validated in extensive cohorts, these models indicate that isolated tumor cells in sentinel nodes hold significant clinical weight in melanoma staging, often predicting recurrence risk when assessed alongside primary tumor characteristics such as Breslow depth [20]. Akkooi et al. analyzed a series of 388 positive SNBs and confirmed that higher tumor burden in the SNB is associated with worse overall survival (OS) and disease-free survival (DFS) [21]. The study showed that patients with micrometastases < 0.1 mm have a significantly better prognosis than patients with metastases > 0.1 mm, and a comparable prognosis to patients with negative lymph nodes [21]. In the study by Madu et al., an SNB metastasis size threshold of 1 mm demonstrated a clear distinction in survival outcomes for stage IIIA melanoma in both the seventh and eighth editions of the staging guidelines. Patients with SN metastases smaller than 1 mm exhibited outstanding distant metastasis-free survival and melanoma-specific survival rates [22]. The study by Verver et al. analyzed the role of SNB micrometastases in patients with CM to better define surgical management and adjuvant therapies. The main results confirm that tumor burden in the sentinel lymph node is a crucial prognostic factor: patients with micrometastases smaller than 1 mm show a significantly better prognosis than those with more extensive metastases [23].

## 5. Conclusions

In conclusion, our findings contribute to the understanding of staging complexities in CM, specifically the prognostic discrepancies observed between stages IIC and IIIA and the potential significance of isolated cells in SNBs. Future research is warranted to confirm the long-term outcomes associated with ITCs in melanoma and to assess whether alternative staging criteria, which consider the metastatic burden rather than solely the presence of neoplastic cells, might better align with patient prognosis. Until such adjustments are widely accepted, clinicians should exercise caution in interpreting ITCs as equivalent to established metastases, particularly in cases of early-stage melanoma, where ITCs may represent a minimal risk factor rather than an indicator of advanced disease.

## Figures and Tables

**Table 1:** Clinicopathological features.

**Stage**	***N***	**%**
pT1b	112	24.2
pT2a	131	28.4
pT2b	34	7.4
pT3a	52	11.3
pT3b	54	11.7
pT4a	24	5.2
pT4b	55	11.8
**Histological type**	***N***	**%**
SSM	342	74.0
NM	105	22.7
Other	15	3.3
**Location**	***N***	**%**
H/N	98	21.2
Trunk	174	37.7
Upper limb	78	16.9
Lower limb	112	24.2

Abbreviations: *N*: number; SSM: superficial spreading melanoma; NM: nodular melanoma; H/N: head and neck.

**Table 2:** SNB assessment according to the stage.

Stage	Positive SNB (*N*; %)	Negative SNB (*N*; %)
pT1b	6; 5.4	106; 94.6
pT2a	21; 16.0	110; 84.0
pT2b	9; 26.5	25; 73.5
pT3a	12; 23.1	40; 76.9
pT3b	20; 37.0	34; 63.0
pT4a	8; 33.3	16; 66.7
pT4b	30; 54.5	25; 45.5

Abbreviation: SNB: sentinel node biopsy; *N*: number.

## Data Availability

The original contributions presented in this study are included in the article. Further inquiries can be directed to the corresponding author.
